# Natural Products in Cancer Research: Mechanistic Advances, Translational Challenges, and the Emerging Role of Chilean Biodiversity

**DOI:** 10.3390/molecules31142469

**Published:** 2026-07-15

**Authors:** Roxana González-Stegmaier, Julia Rubio-Astudillo, Evelyn Silva-Moreno

**Affiliations:** 1 Fundación Arturo López Pérez OECI Cancer Center, Translational Medicine Laboratory, Centro de Investigación e Innovación en Cáncer, Santiago 7500921, Chile; roxana.gonzalez@falp.org; 2 Instituto Ciencias Biomédicas, Universidad Autónoma de Chile, Santiago 7500912, Chile; julia.rubio@cloud.uautonoma.cl

**Keywords:** natural products, phytochemicals, cancer therapy, Chilean endemic plants, immunomodulation

## Abstract

Natural products have long contributed to oncology by providing structurally diverse metabolites and pharmacologically relevant scaffolds. Despite advances in precision oncology, immunotherapy, and targeted therapies, cancer remains a leading cause of morbidity and mortality worldwide. Plant-derived metabolites continue to attract attention due to their ability to modulate key processes such as apoptosis, metabolic adaptation, epigenetic regulation, oxidative stress, inflammation, and tumor microenvironment signaling. However, current evidence is heterogeneous and often based on simplified in vitro models or insufficiently characterized extracts. This review critically distinguishes pharmacologically validated findings from preliminary claims with limited translational relevance. A narrative synthesis of global advances was conducted, focusing on mechanistic pathways, major phytochemical classes, and emerging technologies shaping compound discovery and preclinical validation. The potential contribution of Chilean biodiversity was also examined. Although several Chilean plant species demonstrate cytotoxic, anti-inflammatory, and antioxidant activities relevant to oncology, most findings remain preclinical, limited by inadequate taxonomic identification, lack of phytochemical standardization, scarce in vivo validation, and insufficient pharmacological characterization. The future impact of natural products on oncology will depend on stricter standards for chemical characterization, biological validation, and translational relevance. Chilean biodiversity represents a promising yet largely prospective source for cancer-related drug discovery.

## 1. Introduction

Cancer remains a major global health challenge, with nearly 20 million new cases diagnosed each year and a persistently high mortality rate despite significant progress in prevention, diagnosis, and treatment [1]. Over the past decade, precision oncology, molecularly targeted therapies, and immunotherapeutic strategies have transformed the management of several cancers. However, tumor heterogeneity, adaptive resistance, immune escape, and treatment-related toxicity continue to limit durable benefits in many settings [2].

Within this context, natural products remain highly relevant to oncology. Historically, they have yielded some of the most influential anticancer agents in clinical use, including vincristine, vinblastine, paclitaxel, and camptothecin-derived drugs [3]. Their ongoing importance is based on structural diversity and the ability to interact with multiple biologic targets. Plant-derived metabolites are still studied as modulators of apoptosis, cell-cycle regulation, oxidative stress, inflammatory signaling, angiogenesis, epigenetic remodeling, metabolic adaptation, and tumor-microenvironment interactions [4,5].

Nevertheless, growing enthusiasm for natural products in cancer research has not always been matched by equivalent pharmacological rigor. Much of the current literature still relies on early-stage in vitro observations involving poorly characterized extracts, supraphysiological concentrations, limited dose–response analysis, or mechanistic claims based on a narrow panel of molecular markers [6,7]. A more critical assessment requires distinguishing between clinically validated compounds, robust preclinical leads, and preliminary bioactivity reports with uncertain pharmacology.

At the same time, natural-product research is undergoing significant technological change. High-resolution metabolomics, molecular networking, multi-omics integration, and computational target prediction are improving the identification and characterization of bioactive metabolites [8]. Advanced experimental platforms such as three-dimensional spheroids, organoids, co-culture systems, and organ-on-a-chip models address longstanding limitations of traditional two-dimensional cell culture [9,10]. This evolving landscape is especially important for countries with unique biological resources such as Chile, whose geographic isolation, diverse climate, and high plant endemism offer a distinctive source of botanical diversity with potential biomedical relevance [11].

The purpose of this review is not merely to list reported bioactivities but to critically evaluate the current state of natural product-based cancer research at both the global and local levels. This narrative review was developed through a critical appraisal of landmark reviews and updated with recent original studies retrieved from major biomedical databases (e.g., PubMed, Scopus, and Web of Science). We discuss the principal mechanisms by which plant-derived metabolites may influence cancer biology, the major phytochemical classes driving the field, emerging discovery technologies, the opportunities and challenges associated with Chilean biodiversity, and the key conceptual, methodological, and translational gaps that continue to limit progress in this area.

## 2. Global Advances in Natural Product-Based Cancer Therapy

Natural products continue to hold a unique position in cancer research because of their structural diversity, evolutionary refinement, and broad biological relevance. Their historical contribution to oncology is well established: agents such as paclitaxel, vincristine, and camptothecin-derived drugs illustrate how naturally occurring metabolites can yield clinically effective therapies [3,5]. However, the modern field extends far beyond these landmark examples; plant-derived metabolites are now studied as modulators of proteostatic stress, senescence, metabolism, chromatin state, inflammatory signaling, and immune function within the tumor microenvironment [4,5].

As an overview, plant-derived natural compounds exert anticancer effects through multiple and interconnected biological mechanisms. These compounds influence key cellular processes involved in tumor initiation, progression, and response to therapy, including the regulation of cell survival, stress responses, metabolism, and immune function.

Table 1 summarizes representative examples of these recent mechanistic studies, highlighting the main compounds evaluated, their molecular targets, the signaling pathways involved, and the biological effects observed across different cancer models.

### 2.1. Mechanistic Pathways of Natural Compounds in Cancer Biology

**Figure 1:**
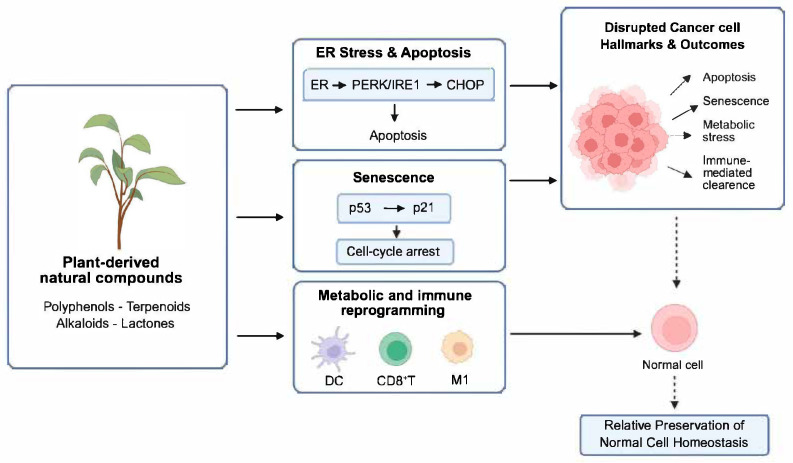
Mechanistic pathways of plant-derived natural compounds in cancer biology. Key mechanisms include ER stress and apoptosis (via PERK/IRE1/CHOP), senescence (p53-p21 pathway leading to cell-cycle arrest), and metabolic and immune reprogramming (involving dendritic cells, CD8^+^ T cells, and M1 macrophages), all contributing to disrupted cancer cell hallmarks and relative preservation of normal cell homeostasis. This image was created at BioRender.com.

A main reason for the ongoing interest in plant-derived metabolites is their ability to influence multiple biological processes simultaneously. Unlike highly selective synthetic inhibitors, many phytochemicals act on interconnected signaling networks, affecting cell survival, inflammation, metabolic adjustment, and stress-response pathways at once. In diseases characterized by redundancy and adaptive plasticity, this multimodal behavior can offer a significant biological advantage. Figure 1 illustrates the key mechanistic pathways by which plant-derived natural compounds modulate cancer biology, including their effects on cell proliferation, apoptosis, inflammation, angiogenesis, metastasis, and immune regulation. However, it also raises the risk of overinterpretation, especially when mechanistic claims are based on a limited set of molecular or phenotype data.

#### 2.1.1. ER Stress and Apoptosis

One of the most common mechanistic themes in natural-product oncology is the induction of endoplasmic reticulum (ER) stress and its progression toward apoptosis. This is biologically plausible because many malignant cells operate near the limits of proteostatic tolerance due to rapid proliferation, increased protein synthesis, hypoxia, and metabolic stress. Under these conditions, disruption of protein folding or ER homeostasis can shift the unfolded protein response (UPR) from an adaptive process to a terminal signaling pathway. The three main UPR branches—PERK-eIF2alpha-CHOP, IRE1-XBP1, and ATF6—are key to this transition and have thus become important mechanistic targets in natural-product research [22,23].

For example, triptolide has been reported to cause sustained ER stress in pancreatic cancer models, with activation of PERK-eIF2alpha and IRE1-XBP1 signaling leading to apoptosis rather than recovery [24]. Flavonoids, terpenoids, and alkaloids have all been linked to ER stress-related apoptosis in cancer cells, although the extent of mechanistic support varies considerably among different compounds and models [22]. However, ER stress in cancer should not be viewed as a universally favorable therapeutic mechanism; its importance depends heavily on the model context, the duration and severity of stress, and whether the observed response truly indicates a targetable vulnerability rather than nonspecific injury [25]. Recent studies have reinforced the mechanistic relevance of ER stress induction by plant-derived metabolites in multiple tumor contexts. Li et al. (2019) demonstrated that icariin, a flavonoid glycoside from *Epimedium* spp., induces PERK-eIF2α-CHOP-dependent apoptosis in triple-negative breast cancer (TNBC) cells (MDA-MB-231 and BT-549) at pharmacologically relevant concentrations (10–40 μM), with selectivity confirmed by comparison against non-tumor MCF-10A cells [12]. Notably, pharmacological inhibition of PERK with GSK2606414 significantly attenuated icariin-induced cell death, providing direct mechanistic evidence for ER stress dependency rather than secondary cytotoxicity. Complementing this, Kamran et al. (2022) reported that thymoquinone (TQ), a bioactive constituent of Nigella sativa, activates the IRE1α–XBP1s axis and promotes apoptosis via sustained ER stress in pancreatic ductal adenocarcinoma (PDAC) organoids derived from patient tissue—a particularly relevant finding given the use of a clinically representative tumor model [13]. These recent data collectively strengthen the mechanistic basis for ER stress-targeting strategies using natural metabolites, while also emphasizing the importance of model selection: organoid and 3D validation could represent a minimum standard for ER stress claims in pharmacologically serious research.

#### 2.1.2. Senescence Induction

Cellular senescence has become a key mechanistic theme because it offers a potential alternative to direct cytotoxicity. Instead of rapidly killing tumor cells, some metabolites may induce a stable state of growth arrest, along with characteristic changes such as altered morphology, senescence-associated beta-galactosidase activity, DNA damage response signaling, and modified telomerase regulation. Recent research has consistently linked several natural metabolites, including baicalein, berberine, and pterostilbene, to this phenotype [26]. The senescence-inducing potential of natural metabolites has been substantially updated in the 2024–2025 literature. Zhang et al. (2025) demonstrated that apigenin (4′,5,7-trihydroxyflavone) could induce oncogene-independent senescence in KRAS-mutated colorectal cancer cells through activation of p21WAF1/CIP1 and concurrent suppression of CDK4/6 activity, with confirmed SASP profiling by multiplex cytokine analysis—a methodological advance over earlier studies that lacked SASP characterization [14]. Critically, the SASP generated in this context was enriched in anti-tumorigenic cytokines (IL-15, IFN-β) rather than pro-inflammatory mediators (IL-6, IL-8), suggesting a more therapeutically favorable senescence phenotype. Additionally, Zhang et al. (2015) showed that a combination of ursolic acid and navitoclax (a senolytic agent) in a sequential treatment protocol effectively eliminated senescent hepatocellular carcinoma cells in vivo, providing early proof-of-concept for ‘induction-then-clearance’ strategies using phytochemical-senolytic combinations [15]. These findings underscore that senescence induction by natural products should now be evaluated not only by classical markers (SA-β-gal, p21, p16) but also by SASP composition and senolytic sensitivity.

In colorectal cancer cells, baicalin was reported to induce a pro-senescent phenotype characterized by reduced hTERT expression, diminished telomerase activity, and features consistent with durable growth arrest, with ERK and p38MAPK signaling involved [27]. In non-small cell lung cancer, pterostilbene was shown to promote senescence through p53-dependent reduction in hTERT and persistent DNA damage response signaling [28]. Berberine has also been associated with senescence-like arrest in glioblastoma models by suppressing EGFR and attenuating the EGFR-MEK-ERK pathway [29]. However, senescence is not inherently beneficial in all circumstances; its significance depends heavily on tumor type, stromal context, immune surveillance, and the composition of the senescence-associated secretory phenotype (SASP) [30].

#### 2.1.3. Metabolic Reprogramming

Metabolic reprogramming has become a key focus of natural product research, especially regarding glycolytic dependence. Polyphenolic metabolites such as resveratrol, quercetin, and epigallocatechin gallate (EGCG) have been repeatedly linked to glucose uptake, lactate production, glycolytic enzymes, and signaling pathways involved in metabolic adaptation, including AMPK, Akt/mTOR, and HIF-1alpha-related pathways [4,31]. Two recent primary studies from 2024 to 2025 substantially advance the mechanistic understanding of natural product-mediated metabolic reprogramming beyond single-enzyme inhibition. Li et al. (2024) performed stable isotope-resolved metabolomics (SIRM) with [U-^13^C]-glucose tracing in EGCG-treated TNBC cells, demonstrating that EGCG does not simply reduce glycolysis but redirects carbon flux from glycolysis toward the pentose phosphate pathway (PPP), increasing ribose-5-phosphate availability and paradoxically supporting nucleotide synthesis under oxidative stress conditions [16]. This nuanced finding cautions against overly simplistic interpretations of reduced lactate as evidence of complete glycolytic inhibition. Separately, Wu et al. (2019) used a comprehensive metabolic flux analysis (MFA) in quercetin-treated hepatocellular carcinoma cells and confirmed that quercetin could simultaneously suppress HK2-dependent glycolysis and glutamine anaplerosis, with both effects mediated through AMPK-dependent phosphorylation of key metabolic enzymes [17]. This dual-pathway suppression provides a more complete picture of metabolic vulnerability exploitation and raises the standard for what constitutes adequate evidence of metabolic reprogramming.

In ovarian cancer cells, resveratrol inhibited glycolysis via AMPK/mTOR-related regulation of metabolic flux while also reducing proliferation [32]. In hepatocellular carcinoma models, quercetin decreased hexokinase 2-dependent glycolysis and dampened Akt-mTOR signaling [17]. In preclinical breast cancer models, EGCG reduced tumor growth in association with decreases in glucose uptake and lactate levels [33]. However, this area is often overstated in the literature; reduced glucose uptake or changes in a single enzyme do not alone prove comprehensive metabolic rewiring. Many studies still lack metabolic flux analyses or broader metabolomic datasets to establish whether tumor cells have genuinely shifted their metabolic state.

#### 2.1.4. Epigenetic Regulation

Epigenetic regulation has become an increasingly important theme because it connects diet-derived and botanical metabolites to reversible mechanisms that influence tumor plasticity and treatment response. The compounds most consistently discussed include curcumin, sulforaphane (SFN), and genistein, all of which have been linked to modulation of DNA methylation, histone deacetylation activity, and non-coding RNA programs [34].

Curcumin has repeatedly been associated with suppression of HDAC activity and increased histone acetylation in cancer cells [35,36]. SFN provides a particularly clear example of promoter-level epigenetic regulation; in human colon cancer cells, it inhibited DNMT1 expression and facilitated demethylation of the Nrf2 (NFE2L2) promoter, enabling the restoration of antioxidant transcriptional programs relevant for chemoprevention [37]. Genistein induced demethylation at CpG sites near the miR-200c/miR-141 loci in prostate cancer cells, supporting the reactivation of EMT-restraining programs linked to epithelial identity [38]. Overall, epigenetic effects induced by natural metabolites should be viewed as biologically informative and potentially relevant for prevention or combination strategies, but not yet sufficient evidence for broad epigenetic normalization in cancer.

Two recent studies significantly expand the epigenetic landscape of natural products in oncology. Li et al. (2020) demonstrated through ChIP-seq and ATAC-seq profiling that sulforaphane (SFN) induces global chromatin remodeling in castration-resistant prostate cancer cells (22Rv1), characterized by increased accessibility at tumor-suppressor gene loci and simultaneous reduction of H3K27me3 marks at key regulatory regions, effects that were dependent on both DNMT3A suppression and EZH2 deacetylation [18]. This genome-wide approach represents a substantial methodological advance over earlier studies relying on single-gene promoter methylation analysis. Furthermore, Özalp et al. (2026) recently reported that berberine modulates the m6A RNA methylation landscape in lung cancer cells by inhibiting the METTL3/METTL14 writer complex, thereby affecting oncogenic mRNA stability of EGFR and MYC transcripts, a finding that expands phytochemical epigenetic activity into the emerging field of epitranscriptomics [19]. This represents a conceptually novel mechanism distinct from traditional DNA methylation or histone modification paradigms and opens new avenues for natural product research in RNA-level regulation.

#### 2.1.5. Immune Modulation

Immune modulation has become a key theme in natural-product oncology, expanding the discussion beyond direct tumor killing to include the tumor immune microenvironment (TIME). Natural products are increasingly studied as immunomodulatory adjuvants capable of influencing antigen presentation, effector lymphocyte activity, myeloid-cell programming, checkpoint pathways, and immunogenic cell death [39]. However, these effects depend heavily on dose, timing, tumor type, and the immune environment under investigation.

Curcumin has been reported to shift Foxp3+ regulatory T cells toward a Th1-like phenotype in lung cancer patients, along with increased IFN-gamma [40]. Resveratrol suppressed tumor growth in a murine renal tumor model in a CD8^+^ T-cell-dependent manner [41]. Curcumin also inhibited the buildup of myeloid-derived suppressor cells (MDSCs) and promoted their differentiation [42]. Withaferin A has been reported to induce immunogenic cell death (ICD) in non-small cell lung cancer cells while increasing PD-L1 expression, further supporting the concept of combining natural-product regimens with immune checkpoint inhibitors [43]. Overall, the current evidence supports certain natural metabolites as promising leads for combination immunotherapy research, but their clinical relevance still requires stronger validation in immunocompetent tumor models.

The immunomodulatory evidence base for natural products has matured considerably in 2024–2026, particularly in the context of combination with immune checkpoint inhibitors (ICI). Miao et al. (2025) demonstrated in an immunocompetent murine CT26 colon tumor model that oral berberine administration enhanced anti-PD-1 efficacy by reducing tumor-infiltrating Foxp3+ Treg cells and increasing the CD8^+^/Treg ratio, effects mediated partly through modulation of the gut microbiome and secondary bile acid metabolism [20]. This is mechanistically important because it links phytochemical administration to systemic immune reprogramming rather than direct tumor cell killing. Complementarily, Amiri et al. (2023) showed that nanoformulated quercetin acts as an immunogenic cell death (ICD) inducer in melanoma models, generating damage-associated molecular patterns (DAMPs: HMGB1, ATP, calreticulin) and potentiating DC maturation in vitro and in vivo, with synergistic antitumor activity observed when combined with anti-CTLA-4 treatment [21]. These findings, conducted in immunologically intact systems, substantially elevate the translational relevance of immune-focused natural product research, though clinical confirmation remains needed.

### 2.2. Key Phytochemical Classes: Structural and Functional Perspectives

Beyond a mechanism-based organization, it is also useful to consider natural products according to their principal phytochemical classes, since structural families often display recurrent pharmacological tendencies as well as characteristic translation limitations. These classes are not homogeneous in their clinical relevance; some include metabolite scaffolds that have already shaped oncology practice, whereas others are composed mainly of compounds that remain mechanistically informative but largely preclinical.

Understanding the strengths and limitations of different phytochemical families is essential for prioritizing candidates with the greatest potential for clinical translation. Table 2 compares the principal classes of natural compounds investigated in cancer research, integrating information on biological activity, pharmacological characteristics, current clinical status, and major development challenges.

#### 2.2.1. Polyphenols and Flavonoids

Polyphenols and flavonoids are among the most thoroughly researched plant metabolites in cancer studies. Resveratrol, quercetin, curcumin, piceatannol, EGCG, and genistein have all been linked to the regulation of inflammatory signaling, apoptosis, epithelial–mesenchymal transition, oxidative stress, and transcriptional programs associated with proliferation and metastasis [4,44,45,46].

Their appeal partly stems from their pleiotropy; resveratrol has been linked to metabolic regulation, inflammatory signaling, and checkpoint-related effects, while quercetin is more specifically associated with glycolytic inhibition and redox modulation [4,17]. Curcumin is discussed in relation to inflammatory, epigenetic, and immune pathways, while EGCG has been connected to both angiogenic and metabolic signaling [4,47]. However, many polyphenols exhibit low oral bioavailability, extensive metabolism, and in vitro activity at concentrations that may not be achievable in the body. Their redox behavior and wide-ranging protein interactions raise concerns about specificity and assay interference. Polyphenols should therefore be viewed as mechanistically rich metabolites whose most practical value may lie in chemopreventive applications, rational combinations, or lead optimization.

Structural features governing the anticancer activity of polyphenols and flavonoids have been systematically examined through SAR studies and computational modeling. For flavonoids, the key pharmacophoric elements include: (1) the 2,3-double bond in the C-ring in combination with the 4-oxo group, which confers planarity and enhances π-stacking interactions with target proteins; (2) the 3′,4′-catechol moiety in the B-ring, critical for radical scavenging, metal chelation (particularly Fe^2+^/Fe^3+^), and interaction with kinase hinge regions; and (3) hydroxyl substitution patterns at C-3, C-5, and C-7 in the A-ring, which modulate both HDAC inhibitory activity and membrane permeability [48]. Quercetin’s higher specificity for PI3K compared to apigenin (which lacks the 3′-OH) illustrates how single hydroxyl group differences translate into meaningful target selectivity [49]. For stilbenes, the trans configuration of resveratrol is essential for biological activity; the cis isomer shows dramatically reduced SIRT1 activation and weaker tubulin interaction. Methylation of the 3,5-hydroxyl groups (as in trimethoxystilbene) improves metabolic stability by preventing O-glucuronidation, the primary inactivation pathway of resveratrol in humans, without eliminating anticancer activity [50]. For Curcumin, the diketone tautomer (predominantly keto form in acidic environments) is considered the active species for Michael addition reactions with cysteine residues in cancer-relevant proteins; the central methylene CH is essential for enone reactivity, though this same property confers PAINS liability. Peracetylation or O-methylation at the phenolic OH groups produce analogs (e.g., EF-24, GO-Y030) with improved metabolic stability and 10–100-fold enhanced antiproliferative potency compared to Curcumin itself [51].

#### 2.2.2. Terpenoids

Terpenoids hold a significant role in oncology, especially because this group includes one of the clearest examples of successful natural-product application. Paclitaxel (Taxol), derived from *Taxus brevifolia*, remains a key chemotherapeutic agent due to its ability to stabilize microtubules, interfere with dynamic remodeling, and trigger mitotic arrest followed by tumor cell death [3,52,53]. This example highlights the clinical importance of terpenoid scaffolds.

Beyond taxanes, research on terpenoids is still largely exploratory. Triterpenoids such as ursolic acid, betulinic acid, and oleanolic acid have been linked to mitochondrial depolarization, cytochrome c release, regulation of Bcl-2 family proteins, inhibition of NF-κB and STAT3, and growth suppression in various tumor models [54,55,56,57,58]. While these compounds demonstrate meaningful biological activity, their development remains limited by poor solubility, unfavorable pharmacokinetics, and the ongoing gap between mechanistic plausibility and consistent therapeutic effectiveness. Terpenoids are therefore best discussed in two categories: taxane-based agents as validated anticancer drugs, and non-taxane terpenoids as promising but predominantly preclinical candidates.

Within the taxane class, the SAR of paclitaxel has been extensively defined: the C-13 side chain (bearing the phenyl-isoserine moiety) is essential for tubulin binding, while the C-2 benzoyl group and the C-4 acetate contribute to binding affinity through hydrophobic contacts within the taxane binding site of β-tubulin. The oxetane ring (D-ring) was historically considered essential for activity but is now known to contribute rigidity rather than direct binding contacts—a finding enabling the design of cyclopropa-taxane analogs with comparable bioactivity [59]. Among triterpenoids, ursolic acid SAR studies indicate that the C-28 carboxyl group is critical for cytotoxicity, while C-3 OH hydroxyl group modifications (esterification, glycosylation) can substantially improve aqueous solubility without abolishing NF-κB inhibitory activity [60]. Betulinic acid C-3 position functionalization with aminoalkyl or fluorinated acyl groups has generated analogs with submicromolar activity in TNBC models, addressing the primary limitation of the parent compound (IC50 typically 5–20 μM) [61]. For sesquiterpene lactones relevant to Chilean biodiversity (see Section 3), the α-methylene-γ-lactone motif is the primary pharmacophore responsible for NF-κB pathway modulation and pro-apoptotic activity through covalent modification of cysteine residues; modifications to this motif (reduction, acetylation) abolish cytotoxic activity while typically preserving anti-inflammatory effects—a selectivity handle potentially relevant for chemoprevention applications [62,63].

#### 2.2.3. Alkaloids and Lactones

Alkaloids and sesquiterpene lactones represent another example of uneven development within a structural class. Camptothecin and its clinically used derivatives remain among the strongest examples of successful natural-product translation, functioning through topoisomerase I (TOP1) poisoning and stabilization of TOP1-DNA cleavage complexes, leading to replication-associated DNA damage and cell-cycle arrest [64,65]. Their clinical relevance is well established and should be clearly distinguished from the more exploratory compounds often grouped within the same category. Berberine illustrates the more experimental side of alkaloid research. It has been linked to AMPK activation, suppression of metastatic behavior, interference with epithelial–mesenchymal transition, and inhibition of proliferative and migratory programs in gastrointestinal and other cancer models [66,67,68]. Sesquiterpene lactones such as parthenolide, artemisinin-related metabolites, and leptocarpin have also garnered attention because of their electrophilic nature and their ability to influence redox-sensitive and inflammatory signaling pathways, especially NF-κB [69,70,71]. While this electrophilicity may be mechanistically beneficial, it also raises concerns about promiscuity and non-specific reactivity.

### 2.3. Technological Advances in Natural-Product Discovery

The current progress in natural-product oncology is closely linked to a new generation of technologies that have transformed the discovery, characterization, and translational development of bioactive metabolites. High-resolution metabolomics, molecular networking, and integrated bioinformatics platforms have significantly improved metabolite annotation, dereplication, and the identification of biologically relevant compounds within highly complex botanical extracts [8,72]. Simultaneously, artificial intelligence (AI) and machine learning approaches are expanding opportunities for target prediction, drug–target interaction modeling, structure–activity relationship analysis, and candidate prioritization, accelerating the transition from natural-product screening to drug development [73]. In parallel, advances in experimental cancer models—including three-dimensional spheroids, patient-derived organoids, co-culture systems, and organ-on-a-chip technologies—provide a more physiologically relevant context for evaluating the anticancer effects of natural compounds. These platforms more accurately reproduce tumor architecture, cellular heterogeneity, microenvironmental gradients, immune interactions, and drug penetration dynamics than conventional two-dimensional cultures, thereby improving the predictive value of preclinical studies and reducing the translational gap between laboratory findings and clinical outcomes [9,10].

Despite these advances, one of the major barriers limiting the clinical application of natural-product-derived therapeutics remains their unfavorable pharmacological properties. Many promising metabolites exhibit poor aqueous solubility, limited chemical stability, rapid systemic clearance, extensive metabolism, and low bioavailability, resulting in insufficient concentrations at tumor sites. Nanotechnology-based delivery systems have emerged as a powerful strategy to overcome these limitations by encapsulating natural compounds within polymeric nanoparticles, lipid-based carriers, liposomes, micelles, solid lipid nanoparticles, nanostructured lipid carriers, or metallic nanomaterials. These platforms not only improve solubility and stability but also prolong circulation time, protect bioactive molecules from degradation, facilitate controlled drug release, and enhance preferential accumulation within tumors through passive and active targeting mechanisms.

**Figure 2:**
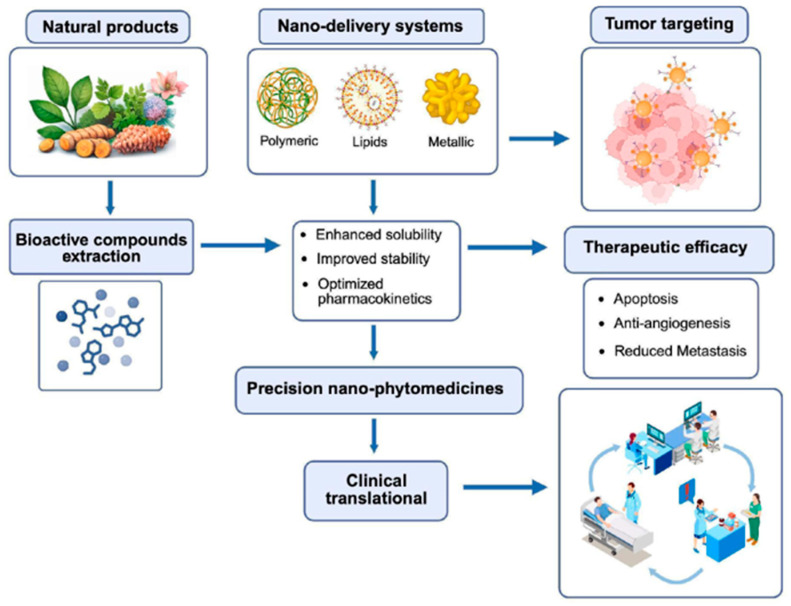
Nano-delivery systems for natural product-based cancer therapy. Bioactive compounds extracted from natural products are incorporated into polymeric, lipid-based, or metallic nano-delivery systems, enhancing solubility, stability, and pharmacokinetics. These precision nano-phytomedicines improve tumor targeting and therapeutic efficacy (apoptosis, anti-angiogenesis, reduced metastasis), support clinical translational applications. This image was created at BioRender.com.

As summarized in Table 3 and Figure 2, an increasing number of nanoformulated natural metabolites have demonstrated superior therapeutic performance compared with their free counterparts across multiple cancer types. Curcumin -loaded polymeric micelles have shown enhanced cellular uptake, increased induction of apoptosis, and greater suppression of colorectal tumor growth in vivo [74]. TPGS-resveratrol solid lipid nanoparticles have exhibited improved intracellular delivery, stronger pro-apoptotic effects, and reduced migratory capacity in paclitaxel-resistant breast cancer cells [75]. Similarly, quercetin-loaded solid lipid nanoparticles have enhanced antiproliferative activity and apoptosis induction in triple-negative breast cancer models [76]. Beyond improving drug delivery, nanoformulations can modulate the tumor microenvironment, inhibit angiogenesis, reduce metastatic dissemination, and increase sensitivity to chemotherapy and immunotherapy. Importantly, the clinical success of albumin-bound paclitaxel (nab-paclitaxel) demonstrates that nanotechnology can facilitate the translation of bioactive compounds into clinically meaningful therapies, leading to improved pharmacokinetics, treatment response, and progression-free survival in patients with metastatic breast cancer [77]. Collectively, these technological and pharmaceutical advances are reshaping the landscape of natural-product-based oncology. The integration of metabolomics, AI-driven discovery platforms, advanced tumor models, and nano-delivery technologies is creating a more robust translational pipeline capable of transforming promising natural metabolites into precision therapeutics. While rigorous biological validation, pharmacological characterization, and well-designed clinical studies remain essential, nano-phytomedicine is increasingly recognized as a critical enabling strategy for unlocking the full therapeutic potential of natural products in cancer prevention and treatment.

#### 2.3.1. Metabolomics and Molecular Networking: Concrete Examples and Success Rates

Despite the widespread adoption of metabolomics-based workflows, converting annotated metabolites into validated anticancer leads remains a significant bottleneck. Feature-based molecular networking and community resources such as GNPS have greatly facilitated metabolite annotation and compound prioritization; however, annotation alone does not establish biological activity. Extensive biochemical, cellular, and in vivo validation is still required before candidate compounds can be considered for translational development [78]. However, taxuyunnanine N, a novel taxane-related—showed preferential cytotoxicity toward taxane-resistant A2780CP ovarian cancer cells in a recent study [79]. Similarly, MZmine3-assisted metabolomics of *Leptocarpha rivularis* (a Chilean native plant discussed in Section 3) enabled more complete annotation of sesquiterpenoid diversity in different plant organs, guiding the isolation of ovatifolin as a second cytotoxic sesquiterpene lactone beyond leptocarpine [80]. These examples illustrate both the practical utility and the stringent downstream validation required to extract pharmacological value from metabolomics-based screens.

#### 2.3.2. Artificial Intelligence and Machine Learning: Validation Gap and Emerging Successes

AI-driven natural product research has substantially accelerated virtual screening, target prediction, and de novo molecular design, expanding the capacity to prioritize candidate molecules for anticancer drug discovery. Nevertheless, current artificial intelligence approaches remain constrained by limitations in training data quality, model interpretability, and generalizability across biological systems. Consequently, experimental validation continues to represent a critical step for confirming the biological relevance and translational potential of AI-generated predictions, underscoring that computational approaches complement rather than replace experimental drug discovery workflows [73]. The development of resveratrol analogs illustrates how rational optimization of natural product scaffolds can enhance biological activity. For example, the synthetic analog (R)-TML104 demonstrated greater biological activity than resveratrol in promoting SIRT1 expression and inhibiting vascular smooth muscle cell phenotypic transformation, ultimately reducing neointima formation in experimental models. These findings highlight the potential of structural optimization to improve the pharmacological performance of natural product derivatives, although further studies are needed to establish their translational value [81]. Recent structural studies have expanded the understanding of druggable conformations in mutant KRAS. Akkapeddi et al. demonstrated that mutant-selective monobody inhibitors can stabilize and probe distinct conformations of the switch II pocket in ***KRAS***(***G12D***), providing experimental evidence that this region represents a tractable target for the rational design of mutation-selective therapeutics. These findings offer a structural framework that may facilitate the future application of computational approaches, including AI-assisted protein modeling and virtual screening, for the discovery of novel KRAS inhibitors [82]. These examples demonstrate that AI tools are most powerful when integrated with rigorous experimental follow-up rather than used as standalone discovery endpoints.

#### 2.3.3. Advanced Experimental Models: Organoids, 3D Systems, and Quantitative Performance Metrics

Three-dimensional tumor models have substantially improved the predictive value of Three-dimensional tumor models are increasingly considered an important platform for improving the translational relevance of preclinical cancer studies, as they better reproduce the structural and functional characteristics of human tumors than conventional monolayer cultures. Despite these advantages, the integration of spheroids, organoids, and other 3D systems into natural product research is still evolving, and many studies continue to employ conventional 2D in vitro models as their primary experimental platform [83]. The increasing use of patient-derived organoids has also expanded opportunities for evaluating natural compounds in biologically relevant tumor models. Compared with conventional two-dimensional cultures, colorectal cancer organoids better preserve tumor heterogeneity and patient-specific drug sensitivity, making them a promising platform for assessing the translational potential of phytochemicals while acknowledging that additional standardization and clinical validation remain necessary before routine application [84]. Similarly, organ-on-a-chip systems that recapitulate tumor vasculature have become an important platform for evaluating the delivery and efficacy of nanomedicines, including natural product-based nanoformulations such as Curcumin. By reproducing key features of the tumor microenvironment under physiologically relevant flow conditions, these models enable more accurate assessment of nanoparticle transport, vascular extravasation, and intratumoral distribution, thereby informing the rational design and optimization of nanoformulations prior to animal studies [85]. These concrete examples illustrate how model sophistication directly influences the pharmacological conclusions drawn from natural product studies.

**Table 3:** Selected nanoformulation natural metabolites in cancer research and translation.

Natural Metabolite	Chemical Structure	Nano-System	Evidence Type	Cancer Model	Main Outcome	Reference
Curcumin	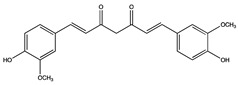	Polymeric micelles	in vitro/in vivo	CT26; subcutaneous CT26 in BALB/c mice	Increased uptake and apoptosis; improved in vivo antitumor efficacy vs. free Curcumin	[74]
Resveratrol	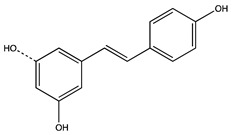	TPGS solid lipid nanoparticles	in vitro/in vivo	SKBR3/PR; xenograft in BALB/c nude mice	Increased uptake, apoptosis; reduced migration/invasion; improved antitumor efficacy	[75]
Quercetin	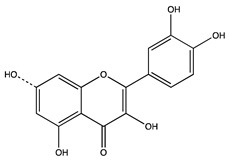	Solid lipid nanoparticles	in vitro	MDA-MB-231; MCF-7	Increased antiproliferative activity and apoptosis; reduced colony formation and angiogenesis	[76]
EGCG	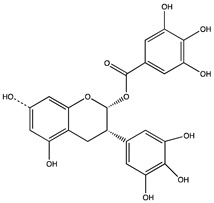	Encapsulated nanoparticles	in vitro	HCT-116, HT-29, HCT-15	Increased uptake and anticancer activity; enhanced ROS-associated damage, DNA fragmentation, and apoptosis	[86]
Genistein	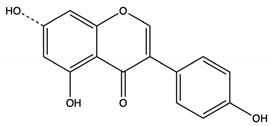	Chitosan nanoparticles	in vitro	HeLa	Increased cellular uptake and anticancer activity	[87]
Paclitaxel	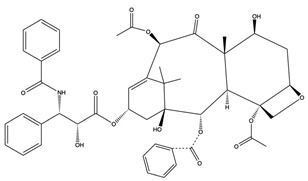	Albumin-bound nanoparticles (nab-paclitaxel)	Clinical study	Taxane-pretreated metastatic breast cancer	Improved clinical activity, PFS, ORR, and disease control	[77]

Abbreviations: CT26, colon tumor 26 murine colon carcinoma cells; TPGS, D-alpha-tocopheryl polyethylene glycol 1000 succinate; SKBR3/PR, paclitaxel-resistant SKBR3 breast cancer cells; BALB/c, Bagg and Albino laboratory-bred mouse strain; MDA-MB-231, triple-negative human breast cancer cells; MCF-7, human breast adenocarcinoma cells; EGCG, (-)-epigallocatechin-3-gallate; ROS, reactive oxygen species; HeLa, human cervical cancer cells; PFS, progression-free survival; ORR, objective response rate.

#### 2.3.4. Critical Evaluation: Translational Bottlenecks Across Technologies

Although significant technological advances have transformed natural-product research in oncology, the translation of promising pre-clinical findings into clinically effective therapies remains limited. High-throughput screening, multi-omics approaches, artificial intelligence, advanced in vitro models, and nanotechnology have substantially improved compound discovery, target identification, and biological characterization. However, each platform faces specific scientific, technical, regulatory, and economic barriers that constrain its impact on successful drug development. Understanding these limitations is essential to avoid overestimating translational potential and to identify areas where methodological improvements are most needed. To provide a balanced perspective, Table 4 summarizes the major technological platforms currently used in natural-product oncology research, highlighting their principal contributions, key bottlenecks, and realistic rates of progression toward clinical application based on the available evidence.

### 2.4. Clinical Evidence for Natural Products in Cancer: Progress, Limits, and Open Questions

Despite the breadth of preclinical data supporting plant-derived metabolites as anticancer agents, their translation into clinical practice has been limited and uneven. As of 2024–2025, clinical trials of natural products in oncology remain largely early-phase, frequently underpowered, and concentrated in a small number of compounds. Nevertheless, a subset of compounds—most notably Curcumin, sulforaphane, and berberine—has progressed to randomized controlled trials (RCTs) with interpretable clinical endpoints, and their emerging results provide important lessons about both the potential and the constraints of natural-product-based oncology [94,95].

#### 2.4.1. Curcumin: Heterogeneous Evidence Across Oncological Indications

Curcumin is the most extensively evaluated phytochemical in cancer clinical trials, with over 290 registered studies documented across oncological and non-oncological indications [96]. A 2024 systematic review that analyzed 34 RCTs involving 2580 patients concluded that curcumin generates heterogeneous results depending on the clinical endpoint and administration context. Significant findings were reported for two specific endpoints: reduction in oral mucositis severity in patients undergoing chemoradiotherapy for head and neck cancer, and attenuation of cancer-associated weight loss [97]. A parallel systematic review and meta-analysis focused specifically on oral mucositis found that curcumin-based formulations reduced the incidence of this toxicity by approximately 6% overall, with a more pronounced 37% reduction observed in patients using curcumin mouthwash during radiotherapy alone [98]. In contrast, neither review identified a meaningful impact of curcumin supplementation on overall survival or progression-free survival, and the evidence for tumor response was inconsistent across indications. A phase IIa open-label RCT (CUFOX) that combined 2 g/day oral curcumin with FOLFOX chemotherapy in 28 patients with metastatic colorectal cancer confirmed acceptable safety and tolerability but did not demonstrate statistically significant improvement in progression-free or overall survival compared with FOLFOX alone [99]. These findings collectively indicate that curcumin, in its conventional oral formulations, may offer clinically meaningful supportive benefits—particularly in managing treatment-related mucositis and preserving body composition—but does not currently show evidence for tumor response as a standalone or adjuvant oncological agent.

A key limitation of curcumin in clinical studies is its poor systemic bioavailability. After conventional oral administration, curcumin typically reaches only low circulating levels because it is poorly absorbed and rapidly metabolized and conjugated [100,101]. This may partly explain why curcumin shows strong effects in preclinical models but has produced modest and inconsistent results in oncology trials [97]. New-generation formulations—including phospholipid complexes, nanomicelles, and solid dispersion systems—are under active evaluation in ongoing trials, but published Phase II data on these delivery platforms in cancer patients remain scarce as of mid-2025. A National Cancer Institute-sponsored Phase II randomized, double-blind, placebo-controlled trial (NCT02782949) evaluated bioavailability-enhanced curcumin (Meriva^®^) for gastric cancer chemoprevention in patients with chronic atrophic gastritis or intestinal metaplasia [102]. Recently published results showed that Meriva^®^ was safe and well tolerated and reduced gastric mucosal IL-1β levels, although histological parameters, DNA-damage endpoints, and several secondary inflammatory markers were similar between treatment arms [103]. This study is among the few controlled assessments of optimized curcumin in a cancer prevention setting to date, and its results are important for evaluating the compound’s clinical utility.

#### 2.4.2. Sulforaphane: Promising Chemopreventive Signals and Consistent Epigenetic Biomarker Modulation

Sulforaphane (SFN), an isothiocyanate derived from glucoraphanin hydrolysis in cruciferous vegetables, has advanced further than most other phytochemicals toward a defined clinical role, particularly in cancer chemoprevention [104,105]. A systematic review published in 2023, which identified eight eligible RCTs in prostate, breast, pancreatic, and melanoma settings, found that SFN produced statistically significant alterations in key epigenetic and proliferative biomarkers, including reductions in HDAC activity and increased Nrf2 target gene expression [105]. However, no study achieved a significant improvement in conventional clinical endpoints such as PSA stabilization or overall survival, and findings on PSA kinetics in prostate cancer patients were inconsistent. A multicenter double-blind RCT involving 78 patients with biochemically recurrent prostate cancer after radical prostatectomy showed that 60 mg/day of stabilized sulforaphane significantly slowed PSA velocity during the 6-month treatment period, with effects that were partially maintained during a subsequent 2-month washout phase, although the study was not powered to assess survival outcomes [106].

More recently, a randomized Phase II trial in former smokers at high risk for lung cancer, demonstrated that 12 months of oral SFN supplementation significantly reduced the bronchial Ki-67 proliferation index compared with placebo (a 20% decrease in the SFN group versus a 65% increase in the control group, *p* = 0.014), with a dose–response relationship between bioavailable SFN and Ki-67 reduction [107]. This finding is particularly notable because Ki-67 reduction in bronchial tissue is considered a validated surrogate endpoint for lung cancer chemoprevention. No severe adverse events were observed in this trial. Taken together, the sulforaphane clinical literature identifies chemopreventive biomarker modulation as the most consistently reproducible signal and supports its further development as a chemopreventive agent in high-risk populations, while underscoring that anti-tumor efficacy in established disease requires more rigorous clinical evaluation.

#### 2.4.3. Berberine: The Strongest Clinical Dataset for a Phytochemical in Cancer Prevention

Among plant-derived alkaloids with cancer-relevant clinical evidence, berberine has accumulated the most compelling dataset, anchored by a rigorously designed multicenter RCT. The Chemoprevention of Berberine in Adenoma Recurrence (CBAR; NCT02226185) was a multicenter, double-blind, randomized placebo-controlled study that enrolled 1108 patients with previous colorectal adenomas after complete polypectomy. Participants received berberine 0.3 g twice daily or placebo for 2 years. In the full analysis set of 891 patients, adenomas recurrence was significantly lower in the berberine group than in the placebo group (36% vs. 47%), and without serious adverse events [108]. A 6-year follow-up extension of the CBAR trial (CBAR-FE, NCT06629051) included 648 participants who underwent at least one colonoscopy during the post-treatment observational period. Adenoma recurrence remained lower in those originally assigned to berberine than in those assigned to placebo (34.7% vs. 52.1%), and total colorectal neoplasm occurrence was also reduced (63.4% vs. 71.0%) [109]. This durability of effect is notable and supports further evaluation of berberine as a candidate for adenoma recurrence prevention.

These findings position Berberine as the most clinically validated phytochemical in colorectal cancer chemoprevention to date and provide a model for how plant-derived alkaloids can progress from mechanistic hypothesis to meaningful clinical evidence through appropriately designed and long-term trials. However, important limitations must be acknowledged: the CBAR evidence is restricted to the chemoprevention of adenoma recurrence and cannot be extrapolated to advanced colorectal cancer treatment or to other tumor types. The mechanisms underlying berberine’s long-term protective effect—potentially involving persistent microbiome modulation, epigenetic reprogramming, or altered stromal signaling—are not fully resolved and represent an important area for future mechanistic investigation.

## 3. Chilean Biodiversity as an Emerging Resource for Anticancer Compounds

Chile provides a biologically unique environment for natural product research because of its clear latitudinal gradient, strong biogeographic isolation, and high level of plant endemism [11,110]. These ecological factors have promoted the diversification of stress-adaptive compounds, phenolics, terpenoids, alkaloids, and sesquiterpene lactones, many of which are now being studied for their antioxidant, anti-inflammatory, cytotoxic, or chemopreventive effects [11,111]. Ethnopharmacological traditions from the Mapuche, Aymara, and Rapa Nui communities further highlight the importance of this biodiversity [112,113].

### 3.1. Species with Documented Anticancer Potential

Among the Chilean taxa currently discussed in oncology-oriented literature, *Leptocarpha rivularis* DC. (*Asteraceae*) is one of the most promising candidates because its evidence goes beyond general antioxidant activity and includes direct observations in tumor models. Its major sesquiterpene lactone, leptocarpine (LTC), has been reported to exert antiproliferative and pro-apoptotic effects in gastric cancer cell lines [71]. Flower extracts obtained with dichloromethane (DCM), ethyl acetate (EtOAc), hexane (Hex), and ethanol (EtOH) were tested in AGS and MKN-45 gastric cancer cells; under the most active conditions, DCM-, EtOAc-, and Hex-derived extracts reduced proliferation, altered cell-cycle distribution, promoted mitochondrial membrane depolarization, increased DEVDase activity, impaired clonogenic capacity, induced senescence, and decreased migration and invasion [71].

Although the available evidence remains predominantly preclinical, several native and endemic plant species have demonstrated promising anticancer activity in cellular and experimental tumor models. Table 5 summarizes the current evidence regarding Chilean plant species investigated for cancer-related applications, including the tested biological material, tumor models evaluated, observed biological effects, and the principal limitations that must be addressed to advance their translational potential.

In micropropagated material, leptocarpine and clonal plant extracts were tested on HeLa cervical cancer cells; after 48 h, leptocarpine at 1–1.2 ppm more effectively reduced metabolic activity in HeLa cells compared to non-tumor CoN cells, and LTC significantly lowered IL-6 and MMP-2 expression [114]. More recently, ovatifolin isolated from the aerial parts of *L. rivularis* was tested on A-2058 and A-375 melanoma cells, revealing live-cell IC50 values of 27.6 and 18.4 microg/mL, respectively [80]. These findings position *L. rivularis* among the most promising Chilean sources of cancer-related sesquiterpenoids, while also highlighting that current evidence remains mainly preclinical and model-dependent.

*Ugni molinae* Turcz. (*Myrtaceae*) has been discussed in cancer-related contexts, although current evidence is more convincing for its antioxidant and anti-inflammatory activities than for direct anticancer effects. Extracts prepared after various drying processes were tested at 0.25 mg/mL for 48 h in NCI-H1975 non-small cell lung cancer cells, with the fresh extract decreasing viability by approximately 13% [115]. Aqueous leaf extracts also reduced viability in AGS gastric adenocarcinoma cells at concentrations from 62.5 microg/mL [116]. The available evidence more consistently supports *U. molinae* as a source of antioxidant and anti-inflammatory metabolites with possible chemopreventive relevance [128,129].

*Berberis microphylla* G.Forst. (*Berberidaceae*) can be more directly linked to cancer-related evidence. Crude and anthocyanin-rich calafate fruit extracts were tested on AGS and G415 gallbladder carcinoma cells; both extracts reduced cell viability and migration, with the anthocyanin-rich extract showing up to 70% inhibition mainly associated with delphinidin derivatives [118].

Beyond these key examples, *Drimys winteri* J.R. Forst. & G. Forst. (*Winteraceae*) and *Peumus boldus* Molina (*Monimiaceae*) highlight why it is important to carefully distinguish between evidence from whole plant species and that from specific metabolites. Ethyl acetate bark extract and isolated drimane sesquiterpenes from *D. winteri* have been tested in human melanoma cells, where they showed anti-growth activity and induced apoptosis, particularly in A375 cells [121]. More recently, essential oil from *D. winteri* leaves reduced proliferation in MCF-7 breast cancer cells at 16–64 μg/mL after 48 h, with no detectable activity at 8 μg/mL. No significant effects were observed in non-tumoral MCF10A mammary cells at 32–64 μg/mL, although the same essential oil also affected non-tumoral HK-2 renal cells while inhibiting the renal tumor lines 786-O and ACHN [122].

In contrast, the available evidence for *P. boldus* centers on boldine, a natural alkaloid found in its leaves and bark. Boldine reduced the viability of the invasive breast cancer cell lines MDA-MB-231 and MDA-MB-468, with 48 h IC50 values of 46.5 ± 3.1 and 50.8 ± 2.7 µg/mL, respectively. Its effects included cytotoxicity, apoptosis, G2/M cell-cycle arrest, disruption of mitochondrial membrane potential, cytochrome c release, activation of caspase-9 and caspase-3/7, inhibition of NF-κB activation, downregulation of Bcl-2 and HSP70, and increased Bax expression [123]. Unlike the evidence described for *D. winteri,* boldine has also been evaluated in vivo. In a rat LA7 mammary adenocarcinoma model, oral administration of boldine at 50 or 100 mg/kg/day reduced tumor volume, whereas the lower dose of 25 mg/kg/day did not. Boldine has also shown chemopreventive activity in a DEN-induced hepatocellular carcinoma model in rats, where treatment was associated with reduced oxidative stress, lower PCNA and Ki67 expression, and changes in key regulators of cell-cycle progression, including p21, p27, Cyclin D1, CDK4, Cyclin E1, and CDK2 [130]. These studies are useful because they show that boldine itself has activity in preclinical models. However, this evidence should not be directly extended to *P. boldus* preparations, unless it is made clear that the data comes from the isolated compound rather than the plant extract.

In the case of *Escallonia* spp. (*Escallonia*ceae), a broad extract-screening study tested 36 preparations from *Escallonia illinita* C.Presl, *Escallonia* rubra (Ruiz & Pav.) Pers., *Escallonia* revoluta (Ruiz & Pav.) Pers., and *Escallonia pulverulenta* (Ruiz & Pav.) Pers. against MCF-7 breast cancer, HT-29 colon adenocarcinoma, and PC-3 prostate cancer cells, using HEK-293T cells as a non-tumor comparison. Among these, the ethyl acetate stem extract of E. rubra was selective for PC-3 cells, with an EC50 of 6.72 μg/mL and a selectivity index of 2.19, while the hexane stem extract of *E. pulverulenta* was selective for HT-29 cells, with an EC50 of 7.52 μg/mL and a selectivity index of 2.31. Further tests at 5, 10, and 25 μg/mL of the extract revealed redox imbalance, increased ROS production, decreased mitochondrial membrane potential, and activation of cell death pathways in the cancer cell lines, suggesting a specific oxidative mechanism rather than broad cytotoxic effects [124].

*Azorella* compacta *Phil*. (*Apiaceae*) has also demonstrated cancer-related activity at both the extract and metabolite levels. A methanolic extract inhibited the growth of several tumor cell lines, including HL60 leukemia, HepG2 hepatocellular carcinoma, SNU-1 gastric carcinoma, MCF-7 breast cancer, HT1080 fibrosarcoma, and A549 lung carcinoma cells, after 24 h of exposure at 1–100 μg/mL. In HL60 cells, the extract showed an IC50 of 34 ± 4.06 μg/mL and induced apoptosis through ROS generation, Bax upregulation, cytochrome c release, caspase-9/3 activation, PARP cleavage, and MAPK-associated signaling [125]. In a complementary phytochemical study, isolated diterpenoids from *A. compacta* were tested against MCF-7 cells, where several compounds reduced viability to below 50% at 100 μM, with *Azorella*ne-type diterpenoids reported as more active than mulinanes [126]. These findings are promising but remain largely limited to early-stage in vitro evidence and still need stronger integration between extract-level and metabolite-level results.

*Lithraea caustica* (Molina) Hook. & Arn. (*Anacardiaceae*) demonstrates a different type of cancer-related evidence. A standardized liter extract (LexT) was tested in a murine B16 melanoma model; both topical treatment and intratumoral injection slowed tumor growth, and topical treatment caused tumor regression in 15% of treated animals, supporting an antitumor immune response rather than a traditional direct cytotoxic mechanism [127]. This highlights that cancer-related activity in Chilean species may also occur through immune-mediated pathways.

### 3.2. Biotechnology, and Sustainability as Enabling Conditions

Biotechnology, chemical standardization, and sustainability should be viewed as essential enabling conditions that influence whether cancer-relevant metabolites can be studied in a reproducible and meaningful translational way. Plant tissue culture and related biotechnological methods are increasingly seen as practical strategies for large-scale production, conservation, and standardization of plant secondary metabolites [131,132].

Among the Chilean examples discussed, *L. rivularis* offers one of the clearest cases of how biotechnology can enhance a pharmacological research process. A micropropagation system allowed for optimizing the introduction, propagation, and rooting stages, with regenerated plants achieving an 83% acclimatization rate under greenhouse conditions [114]. This propagation system was combined with phytochemical and biological analysis, demonstrating that leptocarpine remained the main metabolite and that extracts from clonal plants retained cancer-relevant activity in vitro [114].

In *U. molinae*, different drying methods modified phenolic content along with antioxidant, anti-inflammatory, and tumor-cell viability effects, indicating that pre-analytical processing can influence the apparent pharmacological behavior of an extract before any mechanistic interpretation is made [115]. Sustainable access enhances reproducibility and minimizes ecological impact, but it does not address deeper issues such as dose feasibility, tumor selectivity, pharmacokinetics, or model relevance. These strategies only provide true value when combined with more robust experimental design and more informative biological systems.

Governance and access-and-benefit-sharing also represent important dimensions. In Latin America, access-and-benefit-sharing challenges under the Convention on Biological Diversity and the Nagoya framework have been identified as major barriers to collaborative ethnopharmacological research [133]. Future Chilean natural-product oncology should integrate traceability, community recognition, and fair benefit-sharing into the research process itself, rather than addressing them only as regulatory requirements.

## 4. Gaps, Limitations, and Future Research Priorities

The renewed interest in natural products in oncology reflects a broader shift from reductionist, single-target drug discovery toward a more integrated view of pharmacology. Advances in metabolomics and molecular networking have greatly enhanced dereplication, metabolite annotation, and the understanding of complex botanical mixtures [8,72]. However, these advancements have not addressed a key issue: the evidence supporting many claims of anticancer relevance remains highly inconsistent, often based on simplified in vitro systems or mechanistic interpretations that extend beyond the strength of the available data [134,135].

### 4.1. Preclinical Validation: Methodological Gaps

One of the most ongoing challenges in natural-product pharmacology is the lack of a clear definition of the material being studied. Extract-based studies are still often published without enough information about botanical identity, plant part, extraction solvent, batch consistency, or analytical composition. Current guidance stresses the need for thorough botanical verification, transparent documentation, fit-for-purpose analytical characterization, and batch-to-batch consistency control [134]. Orthogonal analytical methods such as LC-MS/MS and NMR are increasingly seen as critical tools for metabolite fingerprinting and reliable chemical profiling [136,137]. Specifically, reliable phytochemical characterization requires a step-by-step analytical workflow that goes beyond basic extract preparation. HPLC-DAD/PDA fingerprinting is a practical first-line tool for extract profiling, batch comparison, and detection of major components, although it generally does not provide enough structural information to identify individual metabolites in complex mixtures. HRMS and MS/MS analyses offer precise mass measurements, molecular formula assignments, and fragmentation-based annotations, allowing a more detailed assessment of major and minor components [72]. When MS/MS data are analyzed using molecular networks generated with GNPS, chemically related features can be grouped and visualized, facilitating dereplication and the identification of metabolite families inside complex extracts [138,139]. For selected isolated compounds intended for biological testing, qNMR provides an orthogonal approach to confirm content and purity, strengthening the link between chemical identity and biological activity [140]. Overall, HPLC-DAD/PDA, HRMS/MS, GNPS-based networks, and qNMR provide a strong workflow for moving from a crude extract to a chemically characterized lead compound in natural product research with an oncological focus.

Chemical definition, however, is only one part of translational reliability. A large part of the literature still depends on traditional two-dimensional monolayer systems exposed to high and sometimes poorly contextualized concentrations. These assays remain helpful for preliminary screening, but they do not mimic tumor architecture, extracellular matrix constraints, microenvironmental gradients, or adaptive metabolic states that are highly relevant to treatment response [9]. More advanced systems, including organ-on-a-chip platforms, provide a better approximation of tissue complexity and drug-response dynamics [10].

Selectivity and exposure feasibility are additional weaknesses. Many studies evaluate tumor cell lines without including non-tumor comparators or clarifying whether the reported active concentrations are pharmacologically realistic. A reduction in tumor cell viability is not inherently meaningful if it occurs at similarly toxic concentrations in non-malignant cells or at exposure levels unlikely to be achieved in vivo. The interpretation of in vitro antiproliferative activity also depends on the type of material evaluated. Crude plant extracts should not be assessed using the same criteria as isolated compounds because their activity reflects the combined effects of multiple constituents rather than the potency of a single molecule. For this reason, IC₅₀ values for extracts, usually expressed in µg/mL, are best viewed as screening parameters for identifying samples worthy of further fractionation and chemical characterization. In this context, IC₅₀ values below 30 µg/mL are commonly considered promising for crude extracts according to NCI-derived screening criteria [141,142], while values below 20 µg/mL are commonly interpreted as indicative of strong cytotoxic activity in plant-based anticancer studies [142,143]. For isolated and structurally characterized compounds, IC₅₀ values below 10 µM (approximately 4 µg/mL) are generally regarded as indicative of strong cytotoxic activity in early natural-product drug-discovery studies [143]. These cut-offs should be treated as operational rather than universal, since assay type, exposure time, cell seeding density, endpoint definition, tumor cell line, and selectivity against non-tumor cells can substantially influence the magnitude and reproducibility of IC₅₀ values [144,145].

These gaps are worsened by the fact that non-clinical evaluation is not always aligned with internationally recognized safety-testing frameworks such as OECD Good Laboratory Practice [146]. Beyond compliance with OECD Good Laboratory Practices (GLP), botanical pharmacology must be developed within regulatory and analytical standards that support repeatability and translation. The FDA guidance on botanical drug development recognizes the complexity of plant-derived mixtures and emphasizes the need for well-controlled raw materials, chemical characterization, batch consistency, stability data, and a staged non-clinical-to-clinical development plan aligned with IND/NDA requirements [147]. In Europe, the framework of the EMA Herbal Medicinal Products Committee (HMPC) places quality, safety, documented use, and pharmacological plausibility at the center of regulatory assessment and has established approximately 100 community herbal monographs that define current scientific and regulatory standards for herbal substances and preparations used in medicinal products [148].

Many compounds frequently discussed in phytochemical oncology fall into categories associated with pan-assay interference or promiscuous behavior—especially redox-active polyphenols and electrophilic sesquiterpene lactones—whose apparent activity may partly stem from assay interference or non-specific reactivity [149]. Strong in vitro activity should not be equated with translational relevance. The field must shift from solely reporting bioactivity to properly evaluating evidence through a clearer hierarchy supported by appropriate positive and negative controls and non-tumor system comparators.

### 4.2. Challenges and Opportunities in Chilean Biodiversity Research

The case of Chilean biodiversity demonstrates both the potential and the current immaturity of natural-product oncology. Chile has one of the most unique floras in the region, shaped by distinct ecological gradients, geographic isolation, and high endemism [11]. However, most evidence supporting Chilean native or endemic species is still preliminary and scattered, often limited by non-standardized extraction methods, incomplete chemical annotation, weak reproducibility, and limited validation in physiologically relevant tumor models [8,9,11].

The top priority is ensuring taxonomic and chemical accuracy. Full taxonomic validation—including accepted species names, authorities, and families—is crucial for reproducibility, comparability, and regulatory trustworthiness. Current guidelines highlight that botanical authentication and clearly defined analytical preparations are necessary to support meaningful biological interpretation [136]. Without this foundation, cross-laboratory comparisons remain weak and translational extrapolation becomes fragile.

A second priority is advancing the experimental process through a more systematic workflow: starting with taxonomic and phytochemical analysis, then conducting concentration-aware screening in relevant tumor and non-tumor systems, followed by mechanistic studies, validation in three-dimensional or co-culture platforms, and only then moving into justified in vivo models. A third priority focuses on infrastructure and reproducibility through integration of metabolomics-guided annotation, phytochemical traceability, and biologically informative testing [8]. The most valuable investment is probably interdisciplinary: platforms that connect taxonomy, phytochemistry, analytical chemistry, advanced biological models, and early pharmacology.

Ultimately, the future of Chilean biodiversity research depends on clearer prioritization. Not every native or endemic species should be viewed as a potential anticancer agent. A more practical approach would be to differentiate between taxa with specific metabolite-level potential for lead discovery, those with stronger evidence for prevention or anti-inflammatory applications, and taxa whose current value mainly lies in exploratory phytochemistry. The true potential of Chilean biodiversity is not in the immediate development of new anticancer agents, but in the opportunity to establish a more rigorous, taxonomically precise, chemically defined, and translationally cohesive research framework.

## 5. Conclusions

Natural products continue to play a vital role in oncology, not only because they have historically led to some of the most influential anticancer agents in clinical practice, but also because they remain a rich source of chemically diverse metabolites capable of uncovering biologically relevant vulnerabilities in tumor cells and the tumor microenvironment [3,5]. Simultaneously, the current landscape is characterized by a persistent tension between biological potential and scientific rigor. While some compounds and scaffold families have clear pharmacological and translational value, a significant portion of the current literature still relies on preliminary findings based on simplified in vitro systems, incompletely characterized extracts, or mechanistic interpretations that are broader than what the available data can convincingly support.

A key conclusion of this review is that natural products should not be considered a single, uniform therapeutic category. Instead, they should be viewed as a diverse research area that includes clinically validated agents, biologically meaningful lead compounds, prevention-focused metabolites, and many exploratory compounds whose importance remains tentative. Improved standards for taxonomic validation, phytochemical analysis, dose-aware pharmacology, mechanistic understanding, and model selection will be necessary to distinguish truly actionable candidates from metabolites that are simply biologically intriguing [136,149].

In the Chilean context, the true value of biodiversity-based research will only become apparent when biological potential is linked to chemical understanding, sustainability, and institutional governance. Chilean biodiversity should be regarded not as an immediately actionable source of anticancer agents, but as a strategically valuable research platform whose future impact on oncology depends on integrating rigorous pharmacology with ecological and institutional stewardship.

## Figures and Tables

**Table 1:** Molecular mechanisms and biological effects of selected natural compounds in cancer research.

Mechanism	Compound	Cancer Model	Key Finding	Reference
ER Stress & Apoptosis	Icariin (flavonoid)	MDA-MB-231, BT-549 (TNBC); MCF-10A (control)	PERK-eIF2alpha-CHOP-dependent apoptosis at 10–40 µM; selectivity confirmed vs. non-tumor cells	[12]
ER Stress & Apoptosis	Thymoquinone (*Nigella sativa*)	Patient-derived PDAC organoids	IRE1alpha-XBP1s axis activation; sustained ER stress; clinically relevant model	[13]
Senescence Induction	Apigenin (flavone)	KRAS-mutant colorectal cancer cells	p21WAF1/CIP1 activation; CDK4/6 suppression; anti-tumorigenic SASP (IL-15, IFN-beta)	[14]
Senescence Induction	Ursolic acid+ navitoclax	Hepatocellular carcinoma in vivo	‘Induct-then-clear’ strategy; phytochemical-senolytic combination; proof-of-concept	[15]
Metabolic Reprogramming	EGCG	TNBC cells (SIRM with [U-13C]-glucose)	Carbon flux redirected to PPP (not simple glycolysis inhibition); caution against oversimplification	[16]
Metabolic Reprogramming	Quercetin	Hepatocellular carcinoma (MFA)	Dual suppression of glycolysis AND glutamine anaplerosis via AMPK phosphorylation	[17]
Epigenetic Regulation	Sulforaphane (SFN)	22Rv1 castration-resistant prostate cancer (ChIP-seq + ATAC-seq)	Global chromatin remodeling; H3K27me3 reduction; DNMT3A/EZH2 dual suppression	[18]
Epigenetic Regulation	Berberine	Lung cancer cells	METTL3/METTL14 inhibition; m6A epitranscriptomic regulation; EGFR/MYC mRNA destabilization	[19]
Immune Modulation	Berberine (oral)	CT26 murine colon model; anti-PD-1 combination	Enhanced anti-PD-1 via Treg reduction; CD8^+^/Treg ratio increase; gut microbiome-mediated	[20]
Immune Modulation	Nanoformulated quercetin	B16 melanoma (in vivo + anti-CTLA-4)	ICD induction; DAMP generation (HMGB1, ATP, calreticulin); DC maturation; ICI synergy	[21]

Abbreviations: AMPK, AMP-activated protein kinase; ATAC-seq, assay for transposase-accessible chromatin using sequencing; ATP, adenosine triphosphate; CD8^+^, cluster of differentiation 8-positive T lymphocytes; CDK4/6, cyclin-dependent kinases 4 and 6; ChIP-seq, chromatin immunoprecipitation sequencing; CHOP, C/EBP homologous protein; CT26, murine colon carcinoma cell line; DAMPs, damage-associated molecular patterns; DC, dendritic cell; DNMT3A, DNA methyltransferase 3 alpha; EGCG, epigallocatechin-3-gallate; EGFR, epidermal growth factor receptor; ER, endoplasmic reticulum; EZH2, enhancer of zeste homolog 2; HMGB1, high-mobility group box 1; ICD, immunogenic cell death; ICI, immune checkpoint inhibitor; IL, interleukin; KRAS, Kirsten rat sarcoma viral oncogene homolog; m6A, N6-methyladenosine; METTL3/METTL14, methyltransferase-like proteins 3 and 14; MFA, metabolic flux analysis; MYC, MYC proto-oncogene; PD-1, programmed cell death protein 1; PDAC, pancreatic ductal adenocarcinoma; PERK, protein kinase RNA-like endoplasmic reticulum kinase; PPP, pentose phosphate pathway; SASP, senescence-associated secretory phenotype; SIRM, stable isotope-resolved metabolomics; TNBC, triple-negative breast cancer; Treg, regulatory T cell; and XBP1s, spliced X-box binding protein 1.

**Table 2:** Comparative analysis of major phytochemical classes in cancer research: clinical translation, pharmacological properties, and development challenges.

Parameter	Polyphenols/Flavonoids	Terpenoids	Alkaloids & Lactones	Other (Glucosinolates, Lignans)
Clinical approvals (FDA/EMA)	None as isolated phytochemicals	Paclitaxel, Docetaxel, Cabazitaxel	Vinblastine, Vincristine, Irinotecan, Topotecan	None as isolated phytochemicals
Oral bioavailability	Generally low (1–10%); EGCG ~0.1–1.6%; curcumin <1%	Variable; taxanes negligible oral BA; small terpenoids: moderate	Variable; berberine ~5%; colchicine ~45%; camptothecins require prodrug design	Sulforaphane ~80% after hydrolysis; lignans: variable
PAINS/assay interference risk	HIGH: redox-active, Michael acceptors, Fe-chelating (quercetin, EGCG)	MODERATE: electrophilic sesquiterpenes; taxanes: low	MODERATE-HIGH: berberine (fluorescent); electrophilic lactones (parthenolide)	LOW-MODERATE: sulforaphane (reactive thiol); most lignans: low
Synthetic tractability	HIGH: many total syntheses; analog libraries feasible	MODERATE: complex scaffolds; taxane total synthesis achieved but costly	VARIABLE: berberine simple; camptothecin analogs tractable; vinblastine complex	MODERATE: glucosinolate analogs accessible; lignan scaffolds tractable
Dominant mechanistic focus	Apoptosis, epigenetics, metabolic reprogramming, immune modulation	Microtubule dynamics (taxanes); mitochondrial apoptosis (triterpenoids)	Topoisomerase I (camptothecins); AMPK, EMT (berberine); NF-κB (lactones)	Nrf2/ARE response (SFN); ER stress; immune modulation
Current translational stage	Preclinical/Phase I–II (low completion rate)	Taxanes: established clinical use. Non-taxane: early preclinical	Clinically validated (camptothecins, vincas); berberine: Phase II	Chemopreventive trials (SFN in prostate); mostly preclinical
Key delivery challenge	Low solubility, metabolic instability, target promiscuity	Poor aqueous solubility; requires Cremophor or albumin-binding (taxanes)	Systemic toxicity (vincas); poor BA (berberine); instability (camptothecins)	Chemical instability of isothiocyanates; variable food matrix effects

Abbreviations: BA, bioavailability; EGCG, epigallocatechin-3-gallate; EMA, European Medicines Agency; FDA, U.S. Food and Drug Administration; NF-κB, nuclear factor kappa B; Nrf2, nuclear factor erythroid 2-related factor 2; PAINS, pan-assay interference compounds; SFN, sulforaphane; AMPK, AMP-activated protein kinase; EMT, epithelial–mesenchymal transition; ARE, antioxidant response element; ER, endoplasmic reticulum; and Phase I–II, early-stage clinical trials evaluating safety, tolerability, pharmacokinetics, and preliminary efficacy.

**Table 4:** Critical evaluation of some technology platforms in natural product-based cancer research.

Technology Platform	Main Contribution	Major Translational Limitation	Current Evidence Level	Representative Example	Reference
LC–MS/MS and Feature-Based Molecular Networking (GNPS)	Facilitates rapid metabolite dereplication, prioritization of bioactive fractions and annotation of complex extracts.	Most annotations remain putative until confirmed by orthogonal analytical methods (NMR, authentic standards); biological activity still requires experimental validation.	Widely established discovery platform; limited as a stand-alone validation tool.	Annotation of phytochemical families before bioactivity-guided isolation.	[88]
Artificial Intelligence and Machine Learning	Accelerates virtual screening, target prediction and prioritization of natural-product analogs.	Predictive performance depends on training datasets and requires experimental confirmation before translation.	Rapidly expanding; complementary to experimental pharmacology.	AI-assisted prioritization of resveratrol analogs targeting SIRT1.	[89]
Three-Dimensional Spheroids and Patient-Derived Organoids	Better reproduce tumor architecture, cellular heterogeneity and drug penetration than conventional monolayers.	Limited standardization, relatively high cost and incomplete representation of stromal and immune components.	Increasingly accepted preclinical model for precision oncology.	Patient-derived colorectal cancer organoids for therapeutic response assessment.	[90]
Organ-on-a-Chip Systems	Reconstruct dynamic tumor microenvironments including vascular perfusion and tissue–tissue interactions.	Complex fabrication, limited throughput and demanding technical expertise currently restrict widespread adoption.	Highly promising for mechanistic and pharmacokinetic studies.	Tumor-associated vasculature-on-a-chip for evaluating nanoparticle/liposome delivery.	[91]
Stable Isotope-Resolved Metabolomics (SIRM)	Enables direct characterization of metabolic fluxes and pathway utilization following natural-product exposure.	Requires isotope tracers, specialized instrumentation and sophisticated computational analysis.	Valuable mechanistic platform rather than a screening technology.	Carbon flux analysis following EGCG treatment in breast cancer metabolism.	[92]
Nanotechnology-Based Delivery Systems	Improves solubility, bioavailability, pharmacokinetics and tumor accumulation of poorly soluble phytochemicals.	Translation remains constrained by formulation reproducibility, manufacturing scalability and regulatory requirements.	Strong preclinical evidence with limited clinical translation.	Curcumin nanoformulations and lipid-based delivery systems.	[93]

Abbreviations: AI, artificial intelligence; EGCG, epigallocatechin-3-gallate; GNPS, Global Natural Products Social Molecular Networking; LC–MS/MS, liquid chromatography–tandem mass spectrometry; NMR, nuclear magnetic resonance; SAR, structure–activity relationship; SIRM, stable isotope-resolved metabolomics; SIRT1, sirtuin 1.

**Table 5:** Chilean plant species with documented cancer-related activity: summary of tested material, tumor models, biological outcomes, and main evidence limitations.

Species (Authority, Family), Common Name	Tested Material/Major Bioactive Constituents	Tumor Cell Model(s)	Biological Activities/Mechanisms	References
*Leptocarpha rivularis* DC. (*Asteraceae*), palo negro	Flower extracts (DCM, EtOAc, Hex, EtOH); leptocarpine (LTC); ovatifolin	AGS, MKN-45, HeLa, A2058, A375	Antiproliferative and pro-apoptotic effects; mitochondrial depolarization; increased DEVDase activity; senescence; reduced migration/invasion; decreased IL-6 and MMP-2	[71,80,114]
*Ugni molinae* Turcz. (*Myrtaceae*), murta	Berry and aqueous leaf extracts; gastrointestinal digestion products; catechin, pyrogallol, alphitolic, corosolic, and asiatic acids	AGS, Caco-2	Modest tumor-cell viability reduction; antioxidant and anti-inflammatory-associated bioactivity	[115,116,117]
*Berberis microphylla* G.Forst. (*Berberidaceae*), calafate	Crude and anthocyanin-rich fruit extracts; delphinidin derivatives	AGS, G415	Reduced viability and migration; antiproliferative activity associated with anthocyanin-rich extract	[118,119,120]
*Drimys winteri* J.R.Forst. & G.Forst. (*Winteraceae*), canelo	Ethyl acetate bark extract; drimane sesquiterpenes (drimenol, nordrimenone, isonordrimenone, polygodial); leaf essential oil	A375, MCF-7, 786-O, ACHN	Antigrowth and apoptosis-related effects; ROS-associated response; reduced Hsp70 expression; antiproliferative activity in melanoma, breast, and renal tumor cells	[121,122]
*Peumus boldus* Molina (*Monimiaceae*), boldo	Purified boldine	MDA-MB-231, MDA-MB-468	Apoptosis; mitochondrial dysfunction; cytochrome c release; G2/M arrest	[123]
*Escallonia* spp. (*Escallonia*ceae)	Active stem extracts; major compounds not fully resolved	MCF-7, HT-29, PC-3	Selective cytotoxicity; ROS generation; redox imbalance; mitochondrial dysfunction	[124]
*Azorella* compacta Phil. (*Apiaceae*), llareta	Methanolic extract; mulinane and *Azorella*ne diterpenoids	HL60, HepG2, SNU-1, MCF-7, HT1080, A549	Growth inhibition; apoptosis-related signaling; active diterpenoids in breast cancer cells	[125,126]
*Lithraea caustica* (Molina) Hook. & Arn. (*Anacardiaceae*), litre	Standardized Liter extract (LexT)	Murine B16 melanoma model	Delayed tumor growth; topical treatment induced regression in 15% of animals; evidence points to immune-mediated antitumor response	[127]

Abbreviations: DCM, dichloromethane; EtOAc, ethyl acetate; Hex, hexane; EtOH, ethanol; ROS, reactive oxygen species; AGS, human gastric adenocarcinoma cells; MKN-45, human gastric carcinoma cells; HeLa, human cervical adenocarcinoma cells; A2058/A375, human melanoma cells; Caco-2, human colorectal adenocarcinoma cells; G415, human gallbladder carcinoma cells; MCF-7, human breast adenocarcinoma cells; 786-O, human renal cell carcinoma cells; ACHN, human renal adenocarcinoma cells; MDA-MB-231/MDA-MB-468, triple-negative human breast cancer cells; PC-3, human prostate cancer cells; HT-29, human colorectal adenocarcinoma cells; HL60, human promyelocytic leukemia cells; HepG2, human hepatocellular carcinoma cells; SNU-1, human gastric carcinoma cells; HT1080, human fibrosarcoma cells; A549, human lung carcinoma cells; B16, murine melanoma cells; LexT, standardized *Lithraea caustica* extract.

## Data Availability

No new data were created or analyzed in this study. Data sharing is not applicable to this article.

## Abbreviations

The following abbreviations are used in this manuscript:

AMPK	AMP-activated protein kinase
DNMT	DNA methyltransferase
EGCG	Epigallocatechin gallate
EMT	Epithelial–mesenchymal transition
ER	Endoplasmic reticulum
HDAC	Histone deacetylase
ICD	Immunogenic cell death
LTC	Leptocarpine
MDSC	Myeloid-derived suppressor cell
SASP	Senescence-associated secretory phenotype
SFN	Sulforaphane
TAM	Tumor-associated macrophage
TIME	Tumor immune microenvironment
TOP1	Topoisomerase I
UPR	Unfolded protein response
